# Digital Twins in Livestock Farming

**DOI:** 10.3390/ani11041008

**Published:** 2021-04-03

**Authors:** Suresh Neethirajan, Bas Kemp

**Affiliations:** Adaptation Physiology Group, Department of Animal Sciences, Wageningen University & Research, 6700 AH Wageningen, The Netherlands; bas.kemp@wur.nl

**Keywords:** digital twin, precision livestock farming, digitosome, digital cohort, animal farming

## Abstract

**Simple Summary:**

A digital twin can be described as a digital replica of a real-world entity. It simulates the physical state and maybe the biological state and behavior of the real-world entity based on input data. It helps in predicting, optimizing, and improving decision making. It has revolutionized the industrial world, particularly the manufacturing industry, construction and healthcare sector, smart cities, and energy industry. In this perspectives paper, we explore the development and implementation of the digital twin in modern animal farming. In addition to showcasing potential applications, this review provides in-depth insights about the potential implementation and characterization of digital twins in modern animal farming.

**Abstract:**

Artificial intelligence (AI), machine learning (ML) and big data are consistently called upon to analyze and comprehend many facets of modern daily life. AI and ML in particular are widely used in animal husbandry to monitor both the animals and environment around the clock, which leads to a better understanding of animal behavior and distress, disease control and prevention, and effective business decisions for the farmer. One particularly promising area that advances upon AI is digital twin technology, which is currently used to improve efficiencies and reduce costs across multiple industries and sectors. In contrast to a model, a digital twin is a digital replica of a real-world entity that is kept current with a constant influx of data. The application of digital twins within the livestock farming sector is the next frontier and has the potential to be used to improve large-scale precision livestock farming practices, machinery and equipment usage, and the health and well-being of a wide variety of farm animals. The mental and emotional states of animals can be monitored using recognition technology that examines facial features, such as ear postures and eye white regions. Used with modeling, simulation and augmented reality technologies, digital twins can help farmers to build more energy-efficient housing structures, predict heat cycles for breeding, discourage negative behaviors of livestock, and potentially much more. As with all disruptive technological advances, the implementation of digital twin technology will demand a thorough cost and benefit analysis of individual farms. Our goal in this review is to assess the progress toward the use of digital twin technology in livestock farming, with the goal of revolutionizing animal husbandry in the future.

## 1. Introduction

Today, more than ever before, vast quantities of information are being captured, stored, processed, and used digitally. In 1990, before the first internet browser was released by Berners-Lee, less than 0.5% of the world’s population was online. Over the last three decades, the internet exploded, and today, more than half of the world’s population uses the internet [1].

Alternatively, computing power and storage technologies have also increased several folds over the last five decades. To put things into perspective, the latest smartphones that we use typically have 4 GB of RAM. When compared to the Apollo guidance computer [2] on Apollo T human-crewed spacecraft to land on the moon, this is more than one million times its RAM capacity [3]. This explosive growth in computing power, storage capacity and the internet has paved the way for numerous smart devices to exist today. The miniaturization of modern devices/technology has contributed to the advent of the smart devices. In 2020, it is estimated that almost 30 billion smart devices were connected via the internet [4]. This is almost a hundred-fold increase in smart devices since 2006. In other words, we now have almost 26 smart devices per person on this planet, on average.

**Figure 1:**
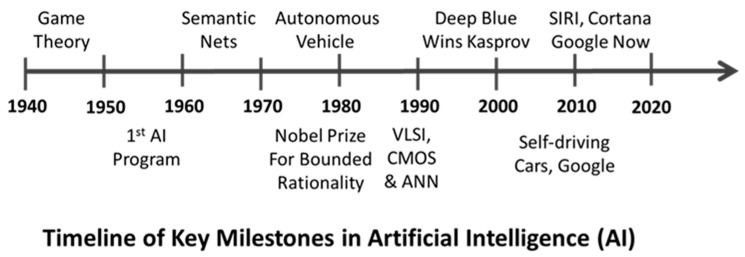
A timeline that shows key milestones in artificial intelligence. VLSI—Very Large-Scale Integration, CMOS—Complementary metal oxide semiconductor, ANN—Artificial Neural Network.

It is estimated that we now generate about 2.5 quintillion bytes of data every day. Clearly, this volume of data is beyond human comprehension. However, newer advances in artificial intelligence (AI), big data and machine learning (ML) have the ability to process such a large volume of data and help us make sense of it (Figure 1). This has opened up new possibilities that never existed before. One such opportunity is digital twins.

This review looks at the concept of digital twins from two perspectives. First, it looks at the origins and practical applications of digital twins that have been adopted across industries and sectors. Secondly, but more importantly, it looks at how digital twins technology can benefit livestock farming in the near future. Traditionally, livestock farming has been a highly experiential and manual industry. Experienced farmers use their knowledge or the knowledge of previous generations to run their operations and care for their livestock. Farming may be more imperfect and unpredictable than other industries, perhaps because it is exposed to the occurrence of weather shocks and pests and diseases. Digital twins promise to revolutionize potentially all aspects of livestock farming. By combining big data, real-time information from the individual farm, and AI, farmers can obtain a much more precise picture of what is occurring with their livestock, housing structures, and equipment. As such, digital twins technology promises to help farmers better predict and discourage negative animal behaviors, track and prevent diseases from spreading or becoming serious, and improve energy efficiency, as well as animal comfort and well-being in housing structures, and reduce the costs of livestock losses and breeding operations.

## 2. The Evolution of Digital Twins

**Figure 2:**
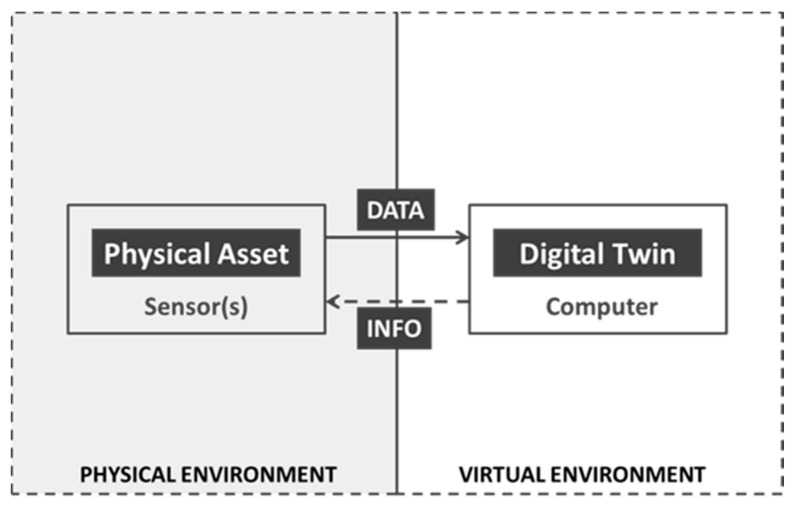
A conceptual representation of digital twin technology and its relation with a physical asset.

At the simplest level, digital twins are realistic virtual representations of a physical entity (Figure 2). This physical entity can be anything from an automobile, windmill, or a manufacturing unit [5]. Sometimes it can even be something as complex as an entire city such as Singapore [6]. To better understand the concept behind digital twins, it is necessary to examine the origins of digital twins and how the concept has evolved to date.

### 2.1. The First Digital Twin

As early as 1993, in his book *Mirror Worlds*, David Gelernter wrote about the possibility of software models that represent some chunk of reality [7]. However, even before that, NASA was one of the first organizations that used complex simulations of spacecrafts [8]. In 1970, the Apollo 13 mission had an unexpected explosion in its oxygen tank [9], which damaged their main engine and pushed the spacecraft away from its trajectory by about 400 miles a minute. To make things worse, the oxygen supply for the crew was slowly leaking into space. However, the mission team quickly modified several high-fidelity simulators to match the real-world conditions of the damaged spacecraft and used this to help the astronauts pick the right moves to land safely back on earth [10]. This was probably one of the first real-world applications of a digital twin.

However, it is important to note that digital twins were not a familiar concept back in 1970. Even so, this specific example met several key characteristics of a digital twin. For instance, the simulators sensed the real-world condition of the spacecraft and used that information to modify themselves. More importantly, it helped the team address what-if scenarios that were never considered in the design plan.

### 2.2. Lower Costs Mean Greater Benefits

Space crafts are extremely costly, mission-critical and inaccessible by anyone not on it. Therefore, in a sense, they were the perfect real-world applications for digital twins—because the high costs were well worth the potential benefits it could offer. At least this was true until a few decades ago. However, as discussed earlier, the cost of sensing, sending, storing, and processing real-world changes in physical entities has become exponentially lower. This opened newer opportunities for several other industries, including the biomedical and agricultural livestock sectors, to also benefit from digital twins.

### 2.3. Early Publications

John Vickers of NASA first coined the term “digital twin” in 2002 [11]. Around the same time, the research professor Dr Michael Grieves worked with Vickers to adapt the concept of digital twins as a way to improve product lifecycle management (PLM) in the manufacturing sector [12]. Initially, he called it the “Conceptual Ideal for PLM”. However, even during this early stage, he touched upon several key properties of digital twins [13]. In his paper, Grieves spoke about the difference between real and virtual spaces and highlighted the need for the exchange of data and information between the real and virtual entities to mirror each other.

### 2.4. The Following Years

Since 2003, interest in the concept of the digital twin has grown by leaps and bounds. Gartner now includes hyper-automation as the number one key strategic technology trend for 2020, and digital twins are a large part of hyper-automation [14]. Initiatives such as Digital Futures and the movement towards the Industry 4.0 paradigm are key factors in this growth of interest. In addition to this, several key advances across technologies, such as the Internet of Things (IoT), big data, and real-time sensors, have driven costs down.

Together, all this has allowed for several new applications of digital twins that were not possible earlier. A range of sensors can now collect data from a smart device and mirror that state in a digital twin in real-time [15]. In other words, we now have the technology to make a reasonably accurate digital twin copy that mimics the properties of real-world assets such as (but not limited to) its shape, status, and movement.

According to Gartner, approximately 75% of the organizations that were implementing IoT projects were already using digital twins [16]. Clearly, the concept is beginning to gain traction, at least among the early adopters. Recent research by Markets and Markets estimated the digital twins market at USD 3.8 billion in 2019. It also projected that this market would grow almost nine-fold to reach USD 35.8 billion in market value by 2025 [17].

### 2.5. Real World Digital Twin Examples

Today, digital twins are being used across sectors and industries in a number of ways, as shown in Table 1.

As one can see from all these examples, there are two common trends. First, digital twins are being applied by industry market leaders across sectors. Secondly, digital twins are being applied in areas that are mission-critical, because they have the potential to improve or transform their market position significantly. This is because digital twin technology is still its nascent stages. In other words, it has high learning, experimentation and implementation costs. These projects often cost millions of dollars per year.

Therefore, naturally, not many companies can afford such a significant investment into something that may not have immediate payoffs, unless of course, they are market leaders and are looking to further consolidate their leadership. In addition, this helps us to better appreciate their big bets. They want their huge investments to pay off with massive returns. This is probably why they are going for the big home runs with the digital twin technology. Does this mean that other smaller companies that cannot innovate with digital twins? What about sectors such as agriculture and livestock production that may not have large R&D budgets? To understand all this better, here is an analysis about what it means to implement digital twins.

## 3. Implementing Digital Twins

### 3.1. Key Properties

As discussed earlier, digital twins are virtual representations of a physical asset. Let us expand on this definition and look at some of the key properties needed to implement a digital twin.

First, to realistically represent a physical asset and mirror its behavior, the twin needs to obtain real-time feedback on how the physical or the biological asset is interacting with its environment, workload and other variables. This requires different sensors that can send and receive specific forms of data via the internet or some other privately secured network.

Second, we need the twin to be able to receive, store and process the large volumes of data in real-time. This requires a significant amount of computing, storage and data processing capacity. In other words, it has to make use of the latest advances in big data, data management and cloud servers.

Third, the twin must be able to make sense of the large volumes of continuously transmitted data. Since this is beyond the computing abilities of most humans, this invariably means using AI algorithms to discern between useful and non-useful information. It also means using AI algorithms to suggest recommendations and actions.

Fourth, the twin must be able to learn about different cause–effect scenarios over time and be able to apply the learnings to improve the performance of the physical asset. This involves running several alternate scenarios, test cases and what-if simulations. Again, this level of complexity is beyond human comprehension. This means that ML algorithms need to be trained under specific circumstances to learn, experiment and evolve the best possible course of action.

**Figure 3:**
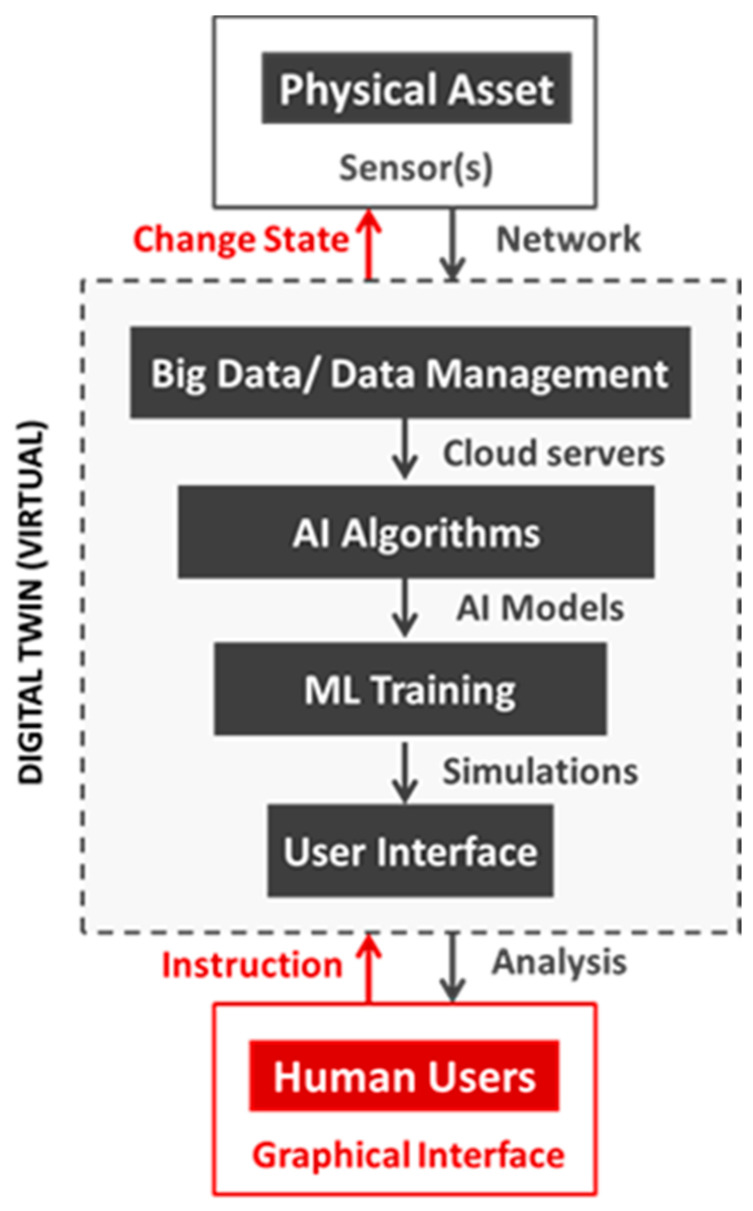
The flow of information between physical asset, digital twin and human users. ML: machine learning.

Finally, all this must be readily available to key human decision-makers via an interactive digital user interface. Typically, this will be some form of a display and processing unit such as computers, tablets or even smartphones (Figure 3).

Key terms involved in the process of implementing digital twins are (1) the actual physical asset, (2) the virtual representation (digital twin), and (3) the human decision-makers.

In addition to this, there are several interconnections, information flows and states that can exist between these three entities. Most of them will vary from project to project, depending upon the scope of the digital twin. Broadly, any digital twin implementation is likely to have the terms described in Table 2.

### 3.2. Beyond Computer Models and Dealing with Uncertainty

Many industries already use computer models and simulations to reduce costs and improve efficiencies. In one sense, digital twins are also simulated computer models. However, there are several differences. The most significant difference is that computer models are built to explore or predict a wide range of cases. For example, we might have a computer model to determine the spread of coccidiosis among farm animals in a particular region. However, a digital twin, by definition, is a virtual representation of a single physical asset. In other words, a digital twin cannot help you make general predictions about a coccidiosis outbreak in a region. Instead, it can only help monitor the key health parameters of Stacy, the dairy cow in farm 1073 at Kentucky.

Thus, the scope of a digital twin is only one individual asset. However, because of this focus, a digital twin is able to go beyond the limitations of most computer models. Digital twins can mirror changes of the physical assets in real-time, with only minor delays ranging between a microsecond to a few minutes. Digital twins also collect and analyze substantially more data compared with most computer models. As a result, digital twins are also able to draw up more realistic what-if scenarios. Dr. Smith recently published an online resource that talked about these key differences (Table 3) [22].

Computer models are known to have gaps in understanding reality. However, a thorough understanding of the data behind them enables the creators to anticipate and correct erroneous results that manifest in the models. They can do so by using several error-correcting algorithms to reduce these errors that stem from the wrong assumptions about the environment. In contrast, a digital twin can potentially capture any data for which a sensor exists as the situation arises and therefore, greatly reduce the degrees of uncertainty.

In addition, unlike most generic computer models, the effective use of a digital twin does not entail an understanding of all the technical details behind its creation. Instead, users can focus all their efforts on learning about how the digital twin behaves under specific conditions. This is much like they would do with any asset in the real world, with almost instant feedback. This is an enormous advantage over generic computer models.

Several research and industry publications have already highlighted the benefits of using digital twins. Jones et al. (2020) performed a systematic literature review and mapped the perceived benefits of using digital twins against the respective publications that described it (Table 4) [23].

## 4. Digital Twins in Livestock Farming

Clearly, there is much to gain from individualized, real-time, actionable feedback from physical assets on a livestock farm. Traditionally, farming has been based on weather and/or price (dynamics) forecasts, the experience of the farmer, and human observations. Digital twins have the potential to radically shift that model, so that future farming is based instead on real-time data, manipulated by AI analyses, which can then fuel better business decisions, improve animal health and well-being, and maximize the return from agricultural resources. An additional benefit is that action can be taken remotely and does not necessitate being at the farm [38].

### 4.1. Precision Livestock Farming (PLF) as a Precursor to Digital Twins

Although digital twin technology in animal husbandry is still in its infancy, the use of precision livestock farming (PLF) has taken advantage of the current technology to improve the management of animal welfare and therefore, the production of animal products.

Increasing demands for automation in the livestock industry coupled with surging labor costs are driving the trend towards PLF. In fact, the market for PLF is expected to grow robustly: from USD 3.1 billion in 2020 to USD 4.8 billion in 2025 [39].

The demonstrated unique ways of solving problems in the animal agriculture industry through PLF opens up pathways for implementing digital twin technology in livestock farms. Wearable animal sensors, a key tool for digital twin technology development, have already been used in PLF as described by Neethirajan and his team [40,41,42,43]. A few examples of the potential of sensor technologies as tools for digital twins in livestock farming are given below.

- Thermal infrared sensors that can measure animal body temperatures by capturing their infrared radiation levels.
- Respiratory rate sensors that typically consist of a belt around the chest (similar to a halter) to measure its thoracic and abdominal movements.
- Immunosensors that can study the saliva and sweat to provide an assessment of hormones, such as cortisol and lactate, in animal biological fluids. This also results in non-invasive tests.
- Photoplethysmography (PPG) uses infrared lights to detect changes in blood volume in the microvascular bed of tissue. It is a non-invasive and cost-effective way to detect blood volume changes.
- A noseband sensor also known as the Rumi Watch that monitors eating and ruminating activities in dairy cows can help farmers to identify and manage stressed animals.
- Water flow sensors can monitor the drinking behavior of large animal herds. They are considered to provide accurate recommendations.
- Accelerometers use electromechanical signals to measure acceleration forces when an animal moves. They have been proven to be highly accurate in monitoring animal activities and movements.
- Pedometers can objectively measure the total number of steps that each animal takes in a day and calculate the total distance it has covered using an algorithm. They can help identify lameness and stress.
- Wireless intraruminal bolus sensors inserted through the esophagus have been developed to monitor the temperature and pH values of the rumen and reticulum. They can help detect diseases such as ruminal acidosis and hypocalcemia.
- Finally, there is the possibility of using these sensors and several more in combination with each other to sense multiple points of stress, disease or physical pain.

Applications of digital twins in livestock farming are beginning to surface and are expected to quickly evolve over the next several years. Consider these promising examples:

### 4.2. Emotional and Mental States of Animals

By using generative adversarial networks machine learning algorithms, farmers can generate real-time, 3D faces of their livestock as a virtual, digital twin. By examining things such as ear position and eye regions present on the virtual model, farmers can better predict animal behavior, anticipate livestock stress, and observe early signs of pain and disease.

### 4.3. Energy Management of a Pigsty

Factors such as temperature, humidity, and ammonia levels can significantly affect the comfort and health of animals feeding indoors. By generating a digital twin of a pigsty before actual construction, farmers can test the effectiveness of windows, fans, and heaters in creating the optimal conditions. Using simulations in EnergyPlus and an actual commercial pigsty in Korea, researchers [44] created a digital twin to determine the most energy-efficient fans to install. Temperature and humidity data were collected at the actual pigsty and used in the simulations. Researchers tested different fan capacities and positions and used the results to select the most energy-efficient, effective solution based on the results.

### 4.4. Monitoring the Movement of Grazing Livestock

By using global positioning systems (GPS) and wireless sensor network (WSN) tracking technology, livestock farmers can not only identify the location of particular animals in a large grazing area but also can observe grazing patterns and behaviors [45]. In addition, if a disease is identified early on, tracking technology allows farmers to easily pinpoint which animals have been in close proximity to sick livestock, preventing the spread of disease and the loss of livestock.

### 4.5. Understanding the Growth and Development of Dairy Animals

Digital twins of dairy cows can be used to better understand the stages of animal growth and development from calf to adult. Multi-agent technology platforms combine sensor and longitudinal data to develop phenotypic traits of animals. The shape, behavior and physiological functions of the animal can be recorded and used for multi-agent planning of animal development and managing life stages and production cycles.

### 4.6. AI-Based Computer Vision to Monitor Livestock

Cainthus, an Irish start-up, uses a smart camera system to monitor animals and operations around the clock. Coupled with advanced AI technology, this camera system translates this real-time, visual data into actionable insights for the farmer to review on a phone, desktop computer, or mobile device. Cargill, an American agricultural company, is partnering with Cainthus to track cattle health.

### 4.7. Augmented Reality Compares Anticipated and Actual Animal Behavior

By observing the activity of pigs and chickens and recording vital signs through sensor technology, farmers can design novel solutions using digital twins to anticipate and prevent damaging behaviors, such as tail biting and feather pecking. Using real-time data and simulations, farmers can predict how pigs and chickens will respond in particular environments, as well as to changes in barns, pens and populations. Augmented reality technology allows comparisons between predicted behavior and actual behavior, providing insight to improve the welfare of livestock.

### 4.8. High-Tech Pedometers Detect Heat Cycles for Breeding

As part of the SmartAgriFood and Fractals accelerator projects [46], digital twins were being used to sense the movements of dairy cows using high-tech pedometers, which is helpful in detecting when a dairy cow is in estrus and ready for breeding. Such monitoring will allow farmers to maximize the efficiency of artificial insemination efforts.

### 4.9. Potential Application Areas

As the costs of creating accurate digital twins reduce even further, there will be opportunities to use this technology to improve PLF practices such as the following:

#### 4.9.1. DATAMATION—Digital Twin Animal Emotions

The emotions of animals can be replicated in digital twins and measured in real time. This enables their incorporation for future real-time animal behavior and disease management predictions. The authors of this paper propose the use of this system of data-based digital twin architecture to predict behavior in animals through measuring emotions from facial features. It will serve as an approach for generating realistic sequences of images and videos of dynamically varying facial expression features of livestock animals from sensor data collected through bio-feedback systems and will be a system for predicting and classifying farm animal emotions using generative adversarial network classifiers.

The deep generative model will allow the synthesis of realistic data samples to enable automated emotion recognition and classification, including cross-modal and unimodal emotion synthesis applications for livestock animals. We propose the use of the data from this real-time biofeedback system (from wearable sensors and camera-based images of farm animals) to determine the animals’ emotions and provide the farmers with emotional feedback through digital twins. This emotion-aware digital twin system is aimed at increasing the welfare of animals through visual feedback and aims to provide critical insights into realistic implementations in enhancing animal welfare.

#### 4.9.2. Gaining Insights on Specific Livestock Conditions

To optimize production, livestock farmers need to ensure that their animals stay healthy. Several parameters, such as pregnancy hormones, body temperature, the quality and quantity of feed intake and the composition of various gases in the animal sheds, can act as reliable indicators.

#### 4.9.3. Detecting the Early Onset of Important Livestock Diseases

More often than not, the naked human eye can only detect diseases after a particular stage. By this time, the disease would have had several opportunities to spread among other animals on the farm, leading to an outbreak. However, digital twins can help farmers detect several important diseases in earlier stages, as well as to isolate exposed livestock from further disease spread.

#### 4.9.4. Optimizing Livestock Feed Intakes

**Figure 4:**
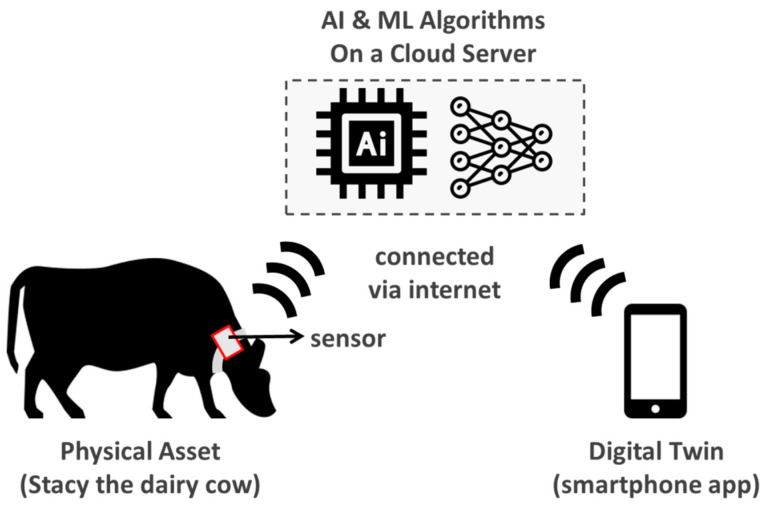
An illustrative case of using digital twin technology to optimize feed intake for a dairy cow.

Owing to the many variables involved, it is almost impossible for farm owners and managers to pinpoint the cause and effect of various feed combinations. However, digital twins can help to simulate changes in feed composition and run what-if scenarios to find out optimal feed intake strategies for a farm (Figure 4).

### 4.10. Limitations of Digital Twins in Livestock Farming

However, this does not mean that there are not any limitations. Next is an analysis of the four largest limitations of using digital twins in the livestock farming sector.

#### 4.10.1. High Switching Costs

Livestock farmers have been optimizing their production practices for centuries together. Most of their best practices come from the careful observation of animals. While new technologies such as digital twins offer new opportunities to care for animals remotely, farmers may be reluctant to change their age-old practices primarily because they have already invested a lot of time, effort, and money into the old way of rearing animals nearby. Asking them to adopt new systems also means that they will have to forego existing labor and processes to an extent. In other words, they face substantial switching costs. This is the most significant limitation in adopting digital twins for livestock farming.

#### 4.10.2. Unknown Risks

High switching costs in technologies for livestock management could be a hindrance for farmers for the adoption of digital twins, which may lead them to take only low-risk decisions. This is because livestock farming has many unknown variables compared with examples such as manufacturing. For example, in manufacturing, most operations take place in a controlled, closed environment. However, livestock farming is dependent on several natural and biological phenomena. Droughts, disease outbreaks, policy changes or even changes in market demand can upset the plans of a farmer in a matter of a few weeks. As you can see, these unknown risks are part and parcel of the farmer’s life. Additionally, these risks are way beyond the scope of influence of the farmer. Therefore, most farmers would also want to adopt new technologies such as digital twins only after they understand how the technology can help to manage such unknown risks. Again, this can be a significant limitation.

#### 4.10.3. Lack of Concrete Evidence

As discussed earlier, several research papers already talk about a plethora of benefits from adopting digital twins (Table 4). However, even among these papers, there are very few examples that validate these benefits. For starters, there is not enough factual evidence available on how digital twins can improve key performance parameters and profits. Even worse, it is difficult to compare these improvements about what is possible without adopting digital twins. It will be a few more years before the technology matures and early experiments start sharing the results. However, the established digital twin models from energy, automotive, human biomedical and manufacturing sectors is expected to provide a framework by characteristics and design methodologies for livestock farming.

#### 4.10.4. Low Return-on-Investment

Adopting new technologies such as digital twins does not come cheap. First, there is the cost of the technology itself. This includes the cost of both hardware and software. Secondly, there are infrastructure costs. This might include the cost of additional energy, internet bandwidth or additional equipment to ensure that the technology can be implemented effectively. Finally, there is a learning cost. This could include training, experimentation, and any losses that farmers might encounter owing to mistakes.

Given all these costs, it might be difficult to justify such substantial changes and expenses without any clarity on how it will pay off. In other words, farmers will continuously evaluate the costs against the potential benefits. Unless this evidence is laid out and the return on investment makes sense, it would be hard to get most farmers to adopt this technology.

#### 4.10.5. Sustainability

One issue that has not been touched upon widely in this review is the sustainability of the livestock industry. While this varies substantially based on the characteristics of individual farms, there have been some consistent problems with the production of livestock, including widespread air and water pollution. Compounds such as airborne ammonia gas and nitrates in ground water can directly threaten human health.

Fortunately, digital twin technology has the potential to help livestock farming become more sustainable over time. For example, Barni et al. (2018) developed a digital twin-based framework built upon system data and processes to assess the sustainability performances of production facilities in enabling sustainability-aware decision making [47].

A 6D building information model was developed using the digital twin processes by adopting and transforming a 3D model of a railway station in England for operating an economic and environmental efficient construction project [48]. The information provided from this railway station project can be adopted and explored for translation into the design of animal husbandry systems and structures for livestock farming. As discussed earlier, Korean researchers used digital twin processes to build a sustainable pigsty [44].

Digital twins can enhance sustainability in a number of ways for livestock farming. By being able to continuously monitor and measure various physiological and vital signs from the animals through wearable sensor technologies realized by the twin, farmers can efficiently manage and even predict diseases even before they occur. This would result in the reduced usage of medicines, antibiotics and other agro-chemicals needed in the livestock sector leading to sustainable animal farming.

In addition, because of the ability of the digital twin to measure the comfort and welfare of animals in real-time, the digital twins, along with the Internet of Things (IoT) can interface in managing the ventilation, heating, feeding stations, milking parlors and fan systems of the animal husbandry and barn/pen structures, leading to the efficient usage of energy management. Digital twins can provide solutions for “stopping dead-on-arrival” for enhancing animal welfare during transportation from the commercial farms to slaughterhouses. During transport, animals are exposed to stressors such as heat stress and cold stress, and crowded spaces leading to aggressive interactions in a short period of time. Sometimes these animals arrive injured or in a state of exhaustion, leading to fatigue or in extreme cases as dead (dead on arrival) to the slaughterhouses. Besides the strong negative effects on animal welfare, this will have negative effects on carcass and meat quality. By being able to measure the welfare of the farm animals during transport using digital twin interfaced sensor technologies, animal welfare can be significantly enhanced, leading to enhanced sustainable conditions for animal transportation.

## 5. Conclusions

The paper takes a balanced approach and acknowledges both the merits of the digital twin technology and simultaneously discusses the existing limitations of adopting this technology from the perspective of livestock farmers. There is a need for more evidence, facts, and case studies of this digital twin technology to encourage widespread adoption among livestock farmers. In the next few years, we expect to see three significant trends. First, we expect to see a significant increase in the number of funded trial projects across the world, to validate the benefits of adopting digital twins in livestock farming. Secondly, we also expect that the primary funding for most of these trial projects will come from governments, research institutions, private investors and even business accelerators. It is quite improbable that the farmers themselves would be willing to pay for these experiments. Finally, we expect that the cost of all the associated technologies required to implement digital twins will continue to decrease even further. This will eventually make this technology increasingly attractive for several specific use cases. We think that this is particularly true in the livestock farming sector as labor costs increase, and opportunities to sustainably run mega-scale livestock operations become more viable. However, we are still at least a few years away from the widespread adoption of digital twins in the livestock farming sector. In the meantime, the three trends discussed above will pave the way for mainstream adoption.

## Figures and Tables

**Table 1:** A list of digital twin case studies across industries and sectors.

Industry	Sector	Digital Twin Types and Advantages	Reference
Boeing	Aero Manufacturing	The digital twin asset development model has shown a 40% quality improvement in first-time parts/systems to deliver enhanced productivity gains.	[18]
Halliburton	Oil Field Service	Using different sensors to capture different dimensions of data while drilling oil wells. Uses this with virtual models to make drilling more efficient.	[19]
Dassault	Software	Using digital twins for various parts of the human body, thus, helping people benefit from less invasive and more personalized medical interventions.	[20]
Unilever	Fast Moving Consumer Goods	Creating virtual models of its factories to track and improve key factory performance parameters and production variables. Helped save USD 2.8 million.	[21]
Royal Dutch Shell	Oil and Gas	Using digital twins to design and recreate realistic real-time models of valuable assets. As a result, are able to reduce maintenance costs, as well as downtime.	[21]
Bridgestone	Tire Manufacturer	Experimenting with real-time data from tire sensors to improve precision safety systems.	[21]

**Table 2:** A summary of key terms used in implementing digital twins.

Key Terms	Description
Physical Environment	Environment where the physical asset exists. Often not easily accessible.
Virtual Simulation	Environment where the virtual digital twin exists. Easily accessible.
Sensory States	Different possible states representing changes in the physical asset.
Changes in State	Switching between various states in the physical asset or digital twin.
Twinning	Synchronization of states between the physical asset and digital twin.
Twinning Rate	The rate at which this synchronization occurs. As close to real-time.
System Processes	Various processes that cause state changes to the asset or twin.

**Table 3:** Dr. Matthew Smith’s INDRA Acronym.

A Digital Twin Needs to Be	
Individual	It must represent a specific thing, e.g., “Daisy the cow” rather than a generic cow.
Near real-time	This also means that the digital twin should be “always on,” available for as long as its real-world counterpart exists.
Data informed	It must be updated via a digital measurement of the real-world thing, e.g., a soil moisture meter or a regular satellite observation.
Realistic	The twin must be a sufficiently realistic surrogate for the real-world thing.
Actionable	Information from the real-world twin must have the potential to lead to an action.

**Table 4:** Characterizing the digital twin: a systematic literature review [23].

Perceived Benefits of Digital Twins—From Characterizing the Digital Twin Research	
Reduces Costs	[24,25,26,27]
Reduces Risks	[27]
Reduces complexity	[28]
Improves after-sales service	[29,30]
Improves efficiency	[31]
Improves maintenance decisions	[32]
Improves security	[33]
Improves safety and reliability	[34]
Improves manufacturing processes	[35,36]
Enhances flexibility and competitiveness	[37]
Fosters innovation	[24]
